# Brain pericytes in culture display diverse morphological and functional phenotypes

**DOI:** 10.1007/s10565-023-09814-9

**Published:** 2023-06-16

**Authors:** Lachlan S. Brown, Natalie E. King, Jo-Maree Courtney, Robert J. Gasperini, Lisa Foa, David W. Howells, Brad A. Sutherland

**Affiliations:** 1 Tasmanian School of Medicine, College of Health and Medicine, University of Tasmania, Level 4 Medical Sciences Precinct, 17 Liverpool St, Hobart, TAS 7000 Australia; 2 School of Psychological Sciences, College of Health and Medicine, University of Tasmania, Hobart, TAS Australia

**Keywords:** Pericytes, Cell culture, Morphology, Contractility, Alpha-smooth muscle actin

## Abstract

Pericytes play several important functions in the neurovascular unit including contractile control of capillaries, maintenance of the BBB, regulation of angiogenesis, and neuroinflammation. There exists a continuum of pericyte subtypes along the vascular tree which exhibit both morphological and transcriptomic differences. While different functions have been associated with the pericyte subtypes in vivo, numerous recent publications have used a primary human brain vascular pericytes (HBVP) cell line where this pericyte heterogeneity has not been considered. Here, we used primary HBVP cultures, high-definition imaging, cell motility tracking, and immunocytochemistry to characterise morphology, protein expression, and contractile behaviour to determine whether heterogeneity of pericytes also exists in cultures. We identified five distinct morphological subtypes that were defined using both qualitative criteria and quantitative shape analysis. The proportion of each subtype present within the culture changed as passage number increased, but pericytes did not change morphological subtype over short time periods. The rate and extent of cellular and membrane motility differed across the subtypes. Immunocytochemistry revealed differential expression of alpha-smooth muscle actin (αSMA) across subtypes. αSMA is essential for cell contractility, and consequently, only subtypes with high αSMA expression contracted in response to physiological vasoconstrictors endothelin-1 (ET1) and noradrenaline (NA). We conclude that there are distinct morphological subtypes in HBVP culture, which display different behaviours. This has significance for the use of HBVP when modelling pericyte physiology in vitro where relevance to in vivo pericyte subtypes along the vascular tree must be considered.

**Graphical abstract:**

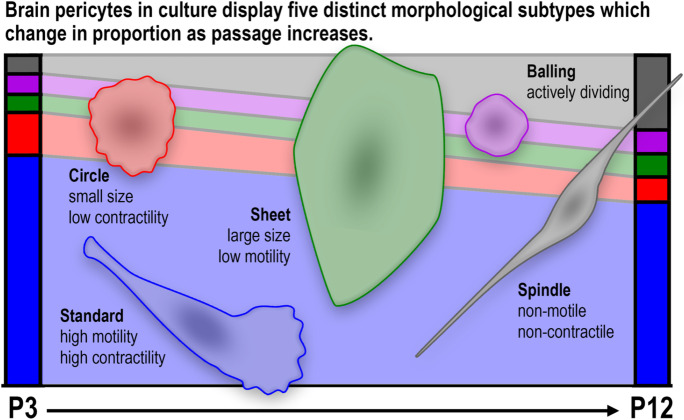

**Supplementary Information:**

The online version contains supplementary material available at 10.1007/s10565-023-09814-9.

## Introduction

Pericytes are a mural cell embedded within the capillary beds of all tissues including the brain. While they were first identified over 150 years ago, many of their functions have only recently been described (Beard et al. 2020; Brown et al. 2019). Pericytes are essential for the maintenance of vascular function, particularly in the brain, where they regulate cerebral blood flow (Hall et al. 2014), maintain the blood–brain barrier (BBB) (Armulik et al. 2010; Bell et al. 2010; Daneman et al. 2010), mediate angiogenesis (Ribatti et al. 2011) and regulate immune cell trafficking and contribute to cytokine and chemokine signalling (Rustenhoven et al. 2017; Stark et al. 2013). Pericyte dysfunction and death have been implicated in the pathophysiology of many neurological diseases including stroke (Hall et al. 2014) and Alzheimer’s disease (Sagare et al. 2013; Sengillo et al. 2013), showing the importance of this cell type to brain function. However, debate in the field continues regarding the exact definition of pericytes (Attwell et al. 2016), particularly due to the significant heterogeneity in morphology and function of pericytes that has been observed at different levels of the vascular tree (Berthiaume et al. 2018b; Grant et al. 2019).

Many advancements in our understanding of pericyte functions have arisen through in vitro studies. While these studies do not incorporate the intact cerebrovascular architecture, cell culture studies provide an excellent method for interrogating the cellular physiology and the effects of stimuli and drugs directly on pericytes. Many approaches exist for isolation and culture of pericytes including primary pericytes isolated from both the rodent brain (Redzic et al. 2015; Tigges et al. 2012) and human brain (Park et al. 2022), as well as pericytes differentiated from human induced pluripotent stem cells (iPSC) (Faal et al. 2019; Gastfriend et al., 2021; Stebbins et al. 2019). Due to the limitations regarding availability, ethics and cost in sourcing primary pericytes and stem cells, as well as technical challenges required to culture them, many researchers are now utilizing commercially available sources of human pericytes, such as human brain vascular pericytes (HBVP) available from ScienCell (USA). HBVP provide a valuable pipeline for translational data given their human origin, the ability to repeat experiments easily, and culturing protocols that are well established. Our analysis of the literature for all primary human brain pericyte culture studies revealed that 64/89 (72%) of the included studies used cells sourced from ScienCell. The widespread use of HBVPs combined with the increase in popularity of pericyte research (Beard et al. 2020) highlights the importance of a comprehensive analysis of morphology and function in this primary pericyte cell source.

Recent papers have reported the use of HBVP to explore a number of pericyte functions including the PDGF-B:PDGFRβ signalling cascade (King et al. 2022; Vanlandewijck et al. 2015), pericyte-mediated vascular inflammation (Navarro et al. 2016) and pericyte contractility in response to vasoactive mediators and ischaemia (Neuhaus et al. 2017). While these functional assays are important, the heterogeneity of pericytes in vitro has recently come to light, which could impact on these functional outcomes. Park et al. (2016) showed phenotypic and functional differences in CD90^+ / −^ subpopulations of pericytes isolated from human brain biopsy tissue, suggesting discrete roles in immune response and scar formation. In cultured pericytes derived from neurologically normal post-mortem cases, there was differential expression of several pericyte markers including alpha-smooth muscle actin (αSMA), PDGFRβ, CD146, NG2, and CD13 (Smyth et al. 2018). Therefore, we wished to determine the morphological heterogeneity of pericytes in a cell culture setting, and whether this was associated with different functions. Specifically, we investigated cell morphology and motility, gene and protein expression, and contractility in HBVP cultures that could have important implications for future studies utilising these widely used primary cells.

## Methods

### *Cell culture*

Primary human brain vascular pericytes (HBVP, #1200) were purchased from ScienCell Research Laboratories (USA), cultured in complete pericyte medium (CPM) (ScienCell, #1201) in a 37 °C incubator in standard conditions (5% CO_2_, 20% O_2_), with media changed every 2–3 days. Cells were cultured and experiments performed between passages 3 and 12, with each passage defined as the period in culture from seeding in a T75 flask with 1 million cells to ~ 90% confluence (~ 4 million cells). Passage intervals varied between 24 and 60 h. To passage, cells were washed in Dulbecco’s Phosphate Buffered Saline (DPBS) − / − (Thermofisher Scientific, #14190144), dissociated from the flask surface with TrypLE (Thermofisher Scientific, #12604013), centrifuged (200 g, 5 min), the supernatant discarded and the pellet resuspended in CPM, cells were counted, and re-plated into new T75 flasks. For experiments, HBVP were grown in T75 flasks to 90% confluence before plating onto glass coverslips (18 mm number 1 glass, Menzel-Glaser) in 12 well plates (1 mL of 5000 cells/mL in each well) for single cell imaging and immunocytochemistry (ICC), and directly into 96 well plates (100 µL of 5000 cells/mL in each well) for multicell imaging.

### *Imaging*

For single cell imaging, high definition differential interference contrast (DIC) microscopy was performed using an upright Nikon Eclipse Ti Live Cell Microscope using a 40 × oil immersion objective with an EMCCD camera (Photometrics Evolve 512 × 512). Glass coverslips (No. 1 18 mm round, Menzel Glaser) incubated in nitric acid overnight (5 M, Merck Millipore) and UV sterilised, were washed, placed in a 12-well plate then coated with poly-L-lysine (0.01% v/v in sterile water, Sigma-Aldrich, Merck, #25988–63-0) for 1.5 h at 37 °C. Coverslips were then rinsed 3 × with CPM and plated with HBVP and allowed to settle overnight until cultures reached approximately 60–80% confluence. HBVP plated on coverslips were transferred to the microscope stage and cells were covered in imaging buffer (HBSS (no Ca^2+^, no Mg^2+^, no phenol red), 15 mM HEPES, 30 mM glucose, 1 mM MgCl_2_, 2 mM CaCl_2_ in Milli-Q H_2_O, pH 7.4) warmed to 37 °C. Cells were maintained at 37 °C and were protected from light. Flow of media was controlled by a gravity pump and vacuum to maintain cellular bathing and enable addition and removal of solutions while imaging. Pericytes were only imaged if there were no other cells in the field of view to allow an accurate determination of each cell’s parameters. For motility and contractility assessment, following baseline images, imaging buffer was removed and replaced with imaging buffer containing vehicle (DMSO, 0.1% v/v), noradrenaline (NA, 30 nM, Sigma #A7257) endothelin-1 (ET1, 50 nM, Sigma #E7764) or adenosine (10 µM, Sigma #A4036). Live DIC images were recorded every 1 min for 20 min. 7–12 cells per coverslip were imaged per session with an automated stage facilitating repeated return to their XY coordinates for recording over time. Images were processed with NIS Elements Analysis software and exported to ImageJ for further analysis.

To enable morphological subtype quantification over a greater number of cells, fixed cells were imaged under phase contrast at 10 × magnification from the centre of each well using a Cytation 5 Cell Imaging Multi-Mode Reader. Images were processed on Gen5 software and exported to ImageJ for further analysis.

To enable quantification of morphology over time, HBVP underwent extended live cell imaging using a temperature and humidity-controlled Nikon Eclipse Ti2-E inverted microscope with a 20 × objective. Cells were imaged every 20 min using DIC mode for 2 h. Images were processed on NIS elements software and exported as 16-bit greyscale TIFF images to ImageJ for further processing.

### *Immunocytochemistry*

Cells were plated on coverslips and allowed to settle overnight. Cells were then fixed (ice cold 100% methanol for 10 min at − 20 °C), permeabilised and blocked with Serum Free Protein Block (Dako, Agilent, #X090930-2) containing Triton X-100 (0.3%v/v, Sigma Aldrich, #1002116296) for 1 h at room temperature. Following washing for 3 × 5 min with PBS (Gibco, #18912014), primary antibody (1:500 rabbit anti-αSMA, Abcam, ab5694) was diluted in Antibody Diluent (Dako, #S080983-2) and applied overnight in a humidified chamber at 4 °C. Cells were then washed for 3 × 5 min with PBS and incubated with 1:1000 donkey anti-rabbit AlexaFluor 488 secondary antibody (ThermoFisher, A-21206) diluted in Antibody Diluent for 1 h at room temperature. Following washing (3 × 5 min PBS then 1 × deionised water) before mounting onto slides using Prolong Gold antifade reagent with 4ʹ,6-diamidino-2-phenylindole (DAPI) mountant (Thermo Fisher, #P36931) for nuclear staining. Slides were imaged on an Olympus FV3000 Super Resolution confocal laser scanning microscope (Olympus, Japan) at 40 × magnification with detector settings as follows: DAPI (Ex. 359, Em. 461), and FITC (Ex. 498, Em. 517). Images were processed on FV-OSR software (Olympus) and exported as raw 16-bit greyscale TIFF images to ImageJ for analysis.

### *Pericyte morphology analysis﻿*

Each individual pericyte was manually assigned to a morphological subtype based on their appearance according to the criteria outlined in Table 1.

**Table 1:** Criteria for categorising individual pericyte morphology from HBVP culture

Morphology	Criteria
Standard	A polarised cell with at least two active distinct membrane projections, one often a larger lamellipodial sheet and the other often a slimmer, longer lamellipodial projection. Nucleus often reaching the edge of the membrane
Circular	Smaller in area than standard pericyte. No distinct primary projections, circular lamellipodial projection surrounding a centralised and often raised nucleus
Sheet	Larger than standard pericyte. Extended, squared, and flattened membrane, flattened central nucleus, often joined to other sheet pericytes
Spindle	Elongated, thin cells with long spindle-like projections of their major membrane processes, compressed and small nuclei. Often joined to other spindle cells in long chains via spindle projections
Balling	Cells appear balled up, are much smaller than standard pericyte, phase bright, and circular

Images of each cell were exported to ImageJ and outlines of cells delineated using the freehand line tool. The following cell parameters were extracted: (1) cell area; (2) cell perimeter; (3) Feret’s diameter (Feret), also known as max caliper, the longest distance between any two points along the selection boundary and is normalised to perimeter, signifying cellular elongation; (4) circularity, calculated using 4π × [Area]/[Perimeter]^2^, with a value of 1.0 indicating a perfect circle and a value approaching 0.0 indicating an increasingly elongated cell shape. For radar plot analysis, quantified data were assigned a score of 1–5 based on their relative correlation (very low, low, moderate, high, very high) respectively to each shape descriptor in comparison to the standard pericyte. Standard pericytes made up the majority of pericytes in the cultures, and were assigned a score of 3 (moderate) for all shape descriptors.

### *Single cell shape analysis over time*

Percentage change in cell morphology (area, perimeter, feret and circularity) was calculated after 20 min relative to baseline using the following formula, where the morphology parameter is referred to as MP:

$$\%\Delta=\frac{\left[MP\left(T=20min\right)-MP\left(T=0min\right)\right]\ast100}{MP\left(T=0min\right)}$$

Positive values represent an increase in the morphology measure compared to baseline and negative values represent a reduction compared to baseline. This analysis was conducted for control cells and for cells exposed to vasoactive agents NA and ET1.

### *Motility analysis﻿*

Cell and process motility were analysed using the MTrackJ plugin for ImageJ (Meijering et al. 2012). Cell motility was measured by tracking the movement of the cell nucleus over time, while cell process motility was analysed by tracking the movement of a primary process and a secondary process on the opposite side of the cell. For circular and sheet pericytes without primary processes, two small projections from the cell body on opposite sides to each other were selected. Velocity was calculated as µm/min. For process velocity, of the two processes, the process with the greatest velocity was used for analysis.

### *αSMA analysis﻿*

For analysis of αSMA expression, cell boundaries were visualised by oversaturated thresholding of the αSMA fluorescent signal. Cell boundaries were traced in ImageJ using the freehand selection tool and each cell was assigned a morphology as per criteria in Table 1. To calculate fluorescent signal per cell normalised for area and background signal, the corrected total cell fluorescence (CTCF) of cellular αSMA immunofluorescence was measured in ImageJ and calculated utilising the below formula as used previously (Jakic et al. 2017):

$$\mathrm{CTCF}=\mathrm{IntDen}(\mathrm{cell})-[\mathrm{area}(\mathrm{cell})\times \mathrm{mean}\;\mathrm{fluorescence}(\mathrm{background})]$$

### *Statistics*

All data were transferred into Microsoft Excel and then imported into GraphPad Prism (Version 9.0) for analysis. When *n* numbers were great enough to generate appropriate power, residuals from each data set were tested for normality using the Shapiro–Wilk normality test when *n* < 50, and Kolmogorov–Smirnov test when *n* > 50. When residuals were normally distributed, data were compared using one-way ANOVA with Dunnett’s multiple comparisons test (comparing to a single control group) or Tukey’s multiple comparisons test (comparing all groups) with a single pooled variance across multiple groups, or with an unpaired *t*-test with Welch’s correction to compare between two groups. When residuals were not normally distributed, data were compared using the non-parametric Kruskal–Wallis test with Dunn’s multiple comparisons test across multiple groups, or with a non-parametric Mann–Whitney or Wilcoxon matched-pairs signed rank test to compare between two groups. All data are presented as mean ± standard deviation (SD). A *p* < 0.05 is considered statistically significant.

## Results

### *Cultured pericytes display five distinct morphological subtypes dependent on passage number*

**Fig. 1:**
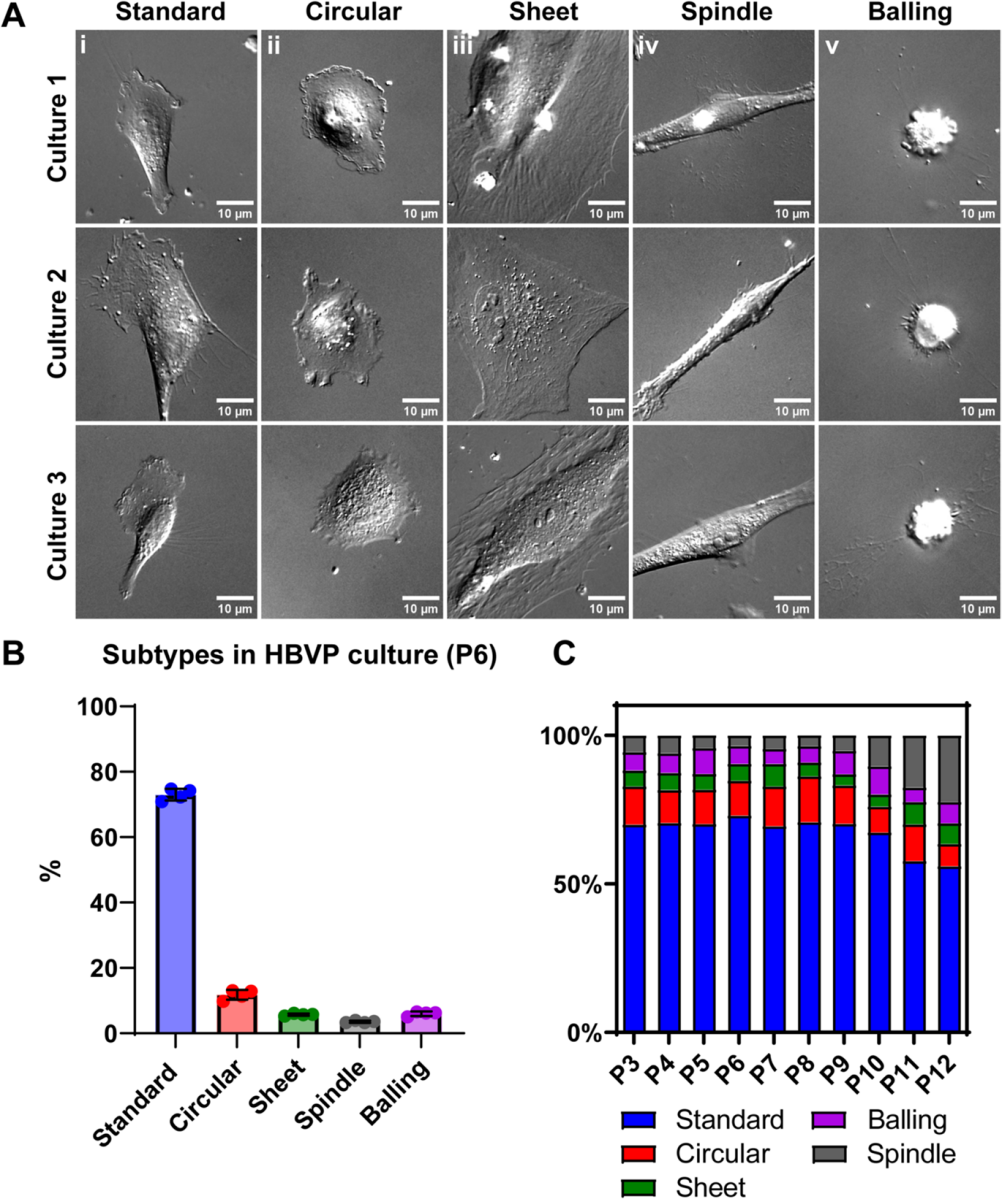
**In vitro pericyte morphological heterogeneity changes with passage number. A** Representative DIC images of five different morphologies observed in three different HBVP cultures: (i) standard, (ii) circular, (iii) sheet, (iv) spindle, and (v) balling morphologies. Images from cultures between P6-P8. Scale = 10 μm. **B** Percentage of each morphology within passage 6 cultures was calculated. *N* = 4 cultures and 360–400 cells analysed per culture (1528 total cells analysed). Data points are presented as individual cultures overlayed with mean ± SD. **C** Percentage of each morphology within cultures was calculated from passages 3–12. *N* = 4 cultures and 583–4625 cells analysed per passage number (20,173 total cells analysed). Residuals were normally distributed, and data for each individual subtype were compared using one-way ANOVA with Dunnett’s multiple comparisons test (comparing back to P3). Error bars are not presented on this figure for clarity

Initially, we wished to determine whether there was heterogeneity in pericyte morphology in culture. Cultured pericytes expressed the canonical pericyte markers including CD13, NG2 and PDGFRβ (Supplementary Fig. 1) as we have described before (King et al. 2022). Pericytes did not express the endothelial marker CD31 (Supplementary Fig. 1). We observed significant heterogeneity in morphology consistently across pericyte cultures (Fig. 1A), which we categorised into five morphologies: standard, circular, sheet, spindle, or balling (Table 1). Pericytes with standard morphology (Fig. 1Ai) often appeared polarised in their membrane projections, migrated readily around the culture well and are consistent with images of pericytes presented previously (Neuhaus et al. 2017). Pericytes with circular morphology (Fig. 1Aii) were less prevalent, appeared to move more slowly and smoothly, and had very active lamellipodial outer membrane movement without major membrane polarisation. Pericytes with sheet morphology (Fig. 1Aiii) appeared to be the largest in size and were much less motile, and often shared plasma membranes with each other. Pericytes with spindle morphology (Fig. 1Aiv) were elongated, had less motility, and appeared to form long-organised chains with other nearby spindle pericytes, similar to the morphology of endothelial cell cultures (Matta et al. 2007). We also observed pericytes with balling morphology (Fig. 1Av) that, following extended observation, 97% underwent cell division within 2 h of balling (Supplementary Fig. 2). In addition, we repeated these experiments in primary rat brain pericytes and a second batch of HBVP cells and showed that these same five morphologies of pericytes were also present in these cultures (Supplementary Fig. 3).

We next assessed the proportion of each pericyte morphological subtype within each culture, and whether this changes as passage number increases. Comparing broadly across passages 3 to 12, the majority of pericytes were always of standard morphology (56–73%, Fig. 1C). Within passage 6 cultures, which were the passage most commonly used in this manuscript, the majority of pericytes consisted of the standard morphology (70.87 ± 1.76%), with circular (11.77 ± 1.49%), sheet (5.70 ± 0.35%), spindle (3.54 ± 0.31%), and balling (6.00 ± 0.69%) making up the remaining cells (Fig. 1B). Similar proportions of each morphology were also observed in primary rat brain pericytes and a second batch of HBVP cells, with the majority of cells exhibiting standard morphology (Supplementary Fig. 3). Comparing across passages from the earliest passage (P3) (Fig. 1C), the proportion of standard morphology pericytes remained consistent up to P10, but was significantly decreased by P11 (P3: 69.97 ± 0.64%; P11: 57.72 ± 2.60%; *p* < 0.0001). Conversely, the proportion of spindle morphology pericytes began significantly increasing by P10 (P3: 5.61 ± 1.13%; P10: 10.37 ± 1.43%; *p* = 0.0092; Fig. 1C). There were no significant differences in proportion of circular, sheet or balling morphologies across any passages (Fig. 1C). In addition, transcriptomic analysis of pericytes at passage 9 compared to passage 5 revealed that there were 96 differentially expressed genes (Supplementary Fig. 4), which may account for changes in the percentage representation of standard and spindle morphology. Among these differentially expressed genes, gene ontology analysis suggests that genes involved in extracellular matrix organization, metabolism and biosynthesis, and symporter activity were most enriched (Supplementary Fig. 5).

### *Morphological subtypes of cultured pericytes possess different shape parameters*

**Fig. 2:**
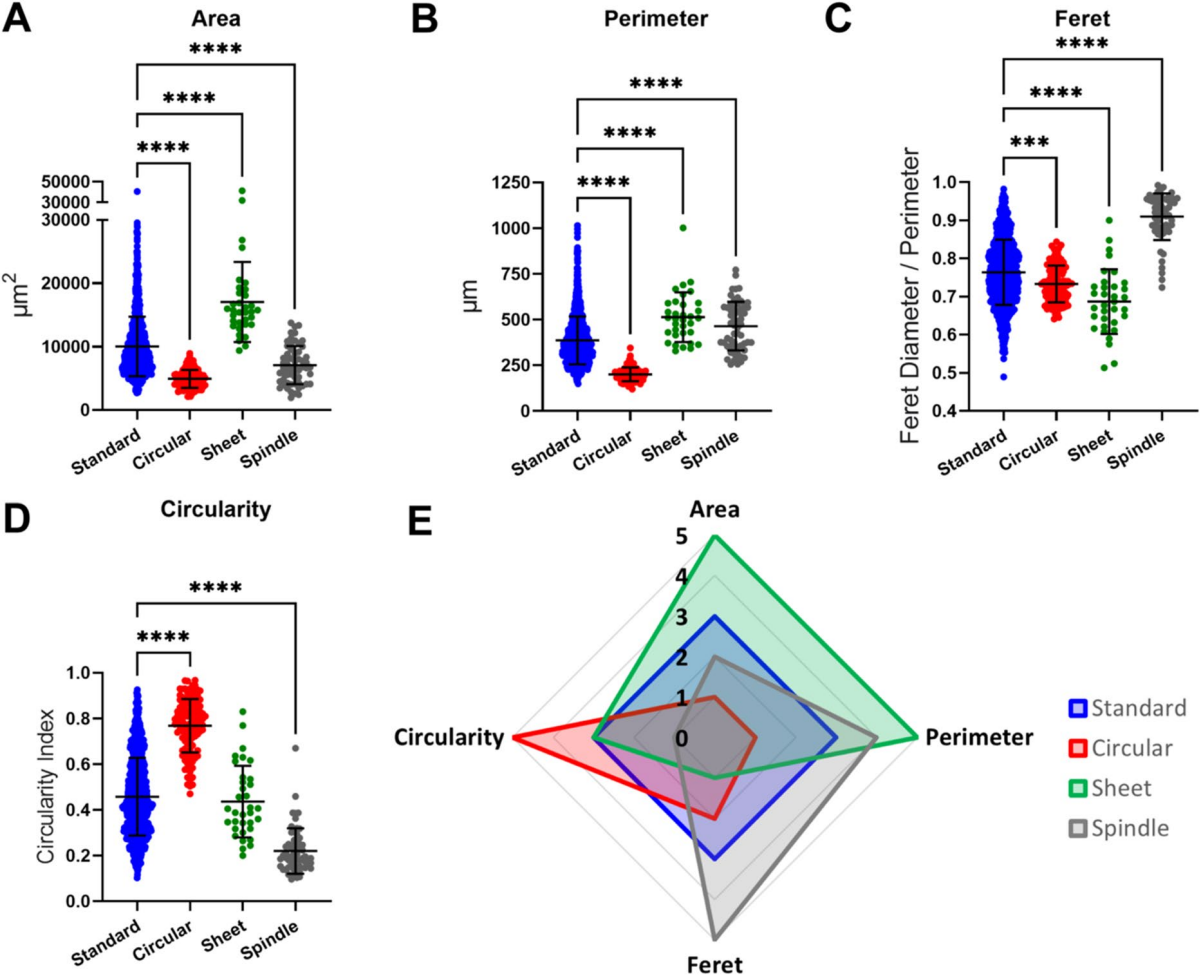
**Morphological subtypes of cultured pericytes possess different shape parameters.** Tracing around each cell provided four different shape parameters that were quantified across different pericyte morphologies. Analysis from *N* = 1219 cells in images of cultures between P6 and P8. (**A**) Cell area was the area (µm^2^) within the traced region of each cell. (**B**) Cell perimeter was calculated as the length (µm) of the trace around each cell. (**C**) Feret’s diameter was determined by the longest distance between any two points along the selection boundary divided by the cell perimeter, an indicator of elongation. (**D**) Circularity Index was defined by 4π × [Area]/[Perimeter]^2^ with a value of 1.0 indicating a perfect circle while a value approaching 0.0 indicating an increasingly elongated shape. Data points are presented as individual cells overlayed with mean ± SD. Kruskal–Wallis test with Dunn’s multiple comparisons test or Ordinary One-Way ANOVA with Dunnett’s multiple comparisons test was used to compare groups relative to standard morphology. *** *p* < 0.001, **** *p* < 0.0001. (**E**) Radar chart describing the relative correlation of each shape descriptor to each morphological subtype. Numbers (1–5) represent very low, low, moderate, high, and very high correlation, respectively

To further characterise the morphological heterogeneity of pericytes in culture, we then quantified four shape parameters for each subtype. Cells exhibiting balling morphology were excluded from this analysis as these cells nearly always went on to divide (Supplementary Fig. 2). Quantitative analysis of cell area (Fig. 2A) revealed that standard pericytes (1.00 × 10^4^ ± 0.47 μm^2^) had a significantly greater cell area than circular (0.49 × 10^4^ ± 0.14 μm^2^, *p* < 0.0001) and spindle (0.71 × 10^4^ ± 0.30 μm^2^, *p* < 0.0001) pericytes, yet significantly smaller than sheet pericytes (1.70 × 10^4^ ± 0.63 μm^2^, *p* < 0.0001). Circular pericytes had the smallest perimeter (Fig. 2B; 201 ± 38 μm), while sheet (513 ± 136 μm) and spindle pericytes (464 ± 133 μm) had the largest, which were all significantly different (*p* < 0.0001) compared to standard pericytes (386 ± 131 μm). Feret’s diameter (Feret), signifying cellular elongation (Fig. 2C), in standard pericytes (0.76 ± 0.09 μm/μm^2^) was significantly larger than circular (0.73 ± 0.05 μm/μm^2^, *p* = 0.0004) and sheet (0.69 ± 0.09 μm/μm^2^, *p* < 0.0001) pericytes, while spindle pericytes (0.91 ± 0.06 μm/μm^2^, *p* < 0.0001) had a significantly larger Feret’s diameter. The circularity (Fig. 2D) of standard (0.46 ± 0.17) and sheet pericytes (0.44 ± 0.16) was similar, while circular pericytes had much greater circularity (0.77 ± 0.12, *p* < 0.0001) and spindle pericytes had much less circularity (0.22 ± 0.10, *p* < 0.0001). The radar plot highlights the consolidation of different shape parameters to enable a quantifiable classification of each morphological subtype (Fig. 2E).

### *Pericyte morphology remains consistent over 2 h*

**Fig. 3:**
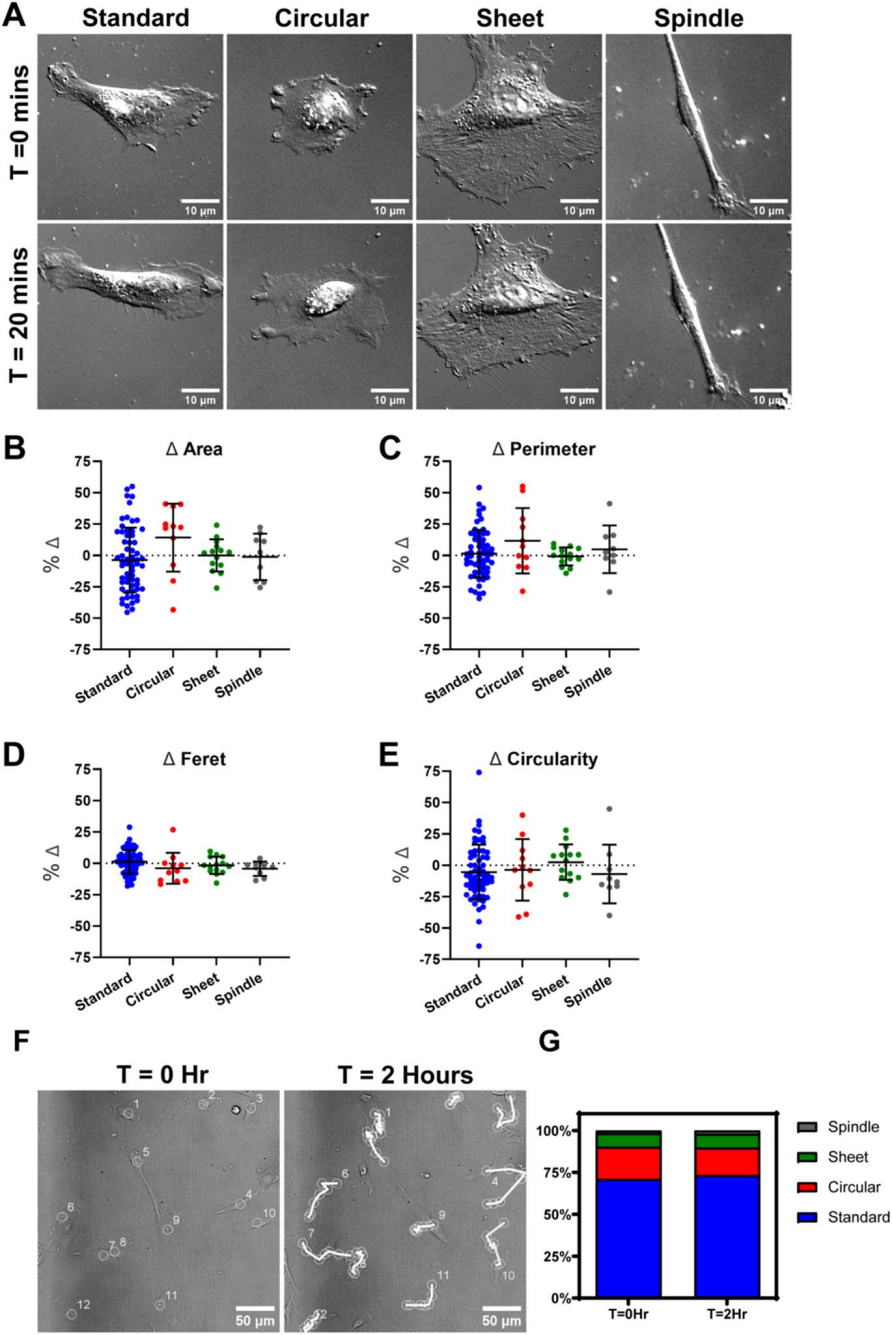
**Pericyte morphology remains consistent but pericytes are motile over 2 h.** (**A**) Representative DIC images showing morphological subtypes at T = 0 and T = 20 min. Images from cultures between P6 and P8. Scale = 10 μm. (**B**–**E**) The change in cell area, perimeter, feret, and circularity of each pericyte subtype was calculated over 20 min. Data points are presented as individual cells overlayed with mean ± SD. Kruskal–Wallis test with Dunn’s multiple comparison test (**B**, **C**, and **E**) or Ordinary One-Way ANOVA with Dunnett’s multiple comparisons test (**D**) was used to compare groups. 96 cells were analysed in total. (**F**) Representative DIC images showing pericytes at T = 0 Hr (i) and T = 2 Hr (ii). Scale = 20 μm. White tracks indicate movement of individual pericytes at 20 min intervals over a 2 h period. Images from cultures between P6 and P8. (**G**) Percentage of four pericyte morphologies (standard, circular, sheet, and spindle) at baseline (T = 0 h) and 2 h (T = 2 h) calculated from DIC images of P6–P8 cultures. *N* = 256 cells analysed

Given that the proportion of pericytes with standard and spindle morphology alters between passages, we next investigated whether pericyte morphological subtypes changed over a shorter time period. Initially, we analysed pericyte morphology in live cultures over 20 min. No changes in morphology including cell area, cell perimeter, Feret’s diameter or circularity were observed for any pericyte subtype over 20 min (Fig. 3A–E). We then extended our observation time to 2 h and assessed individual pericyte morphology. During this time, pericytes were motile and migrated across the well (Fig. 3F). We did notice some small changes between subtypes of cells, e.g. a pericyte would appear to have circular morphology but cell tracking showed that the cell was initially bunched but then spread out to reveal standard morphology. Thus, it is possible that some morphological changes occur during migration. However, when we calculated the proportions of each subtype, there were no differences in overall proportions of each subtype (Fig. 3G). This suggests that pericytes do not change their morphology in a substantial way over a short time period under basal conditions.

### *Pericyte motility and process movement differs across morphological subtype*

**Fig. 4:**
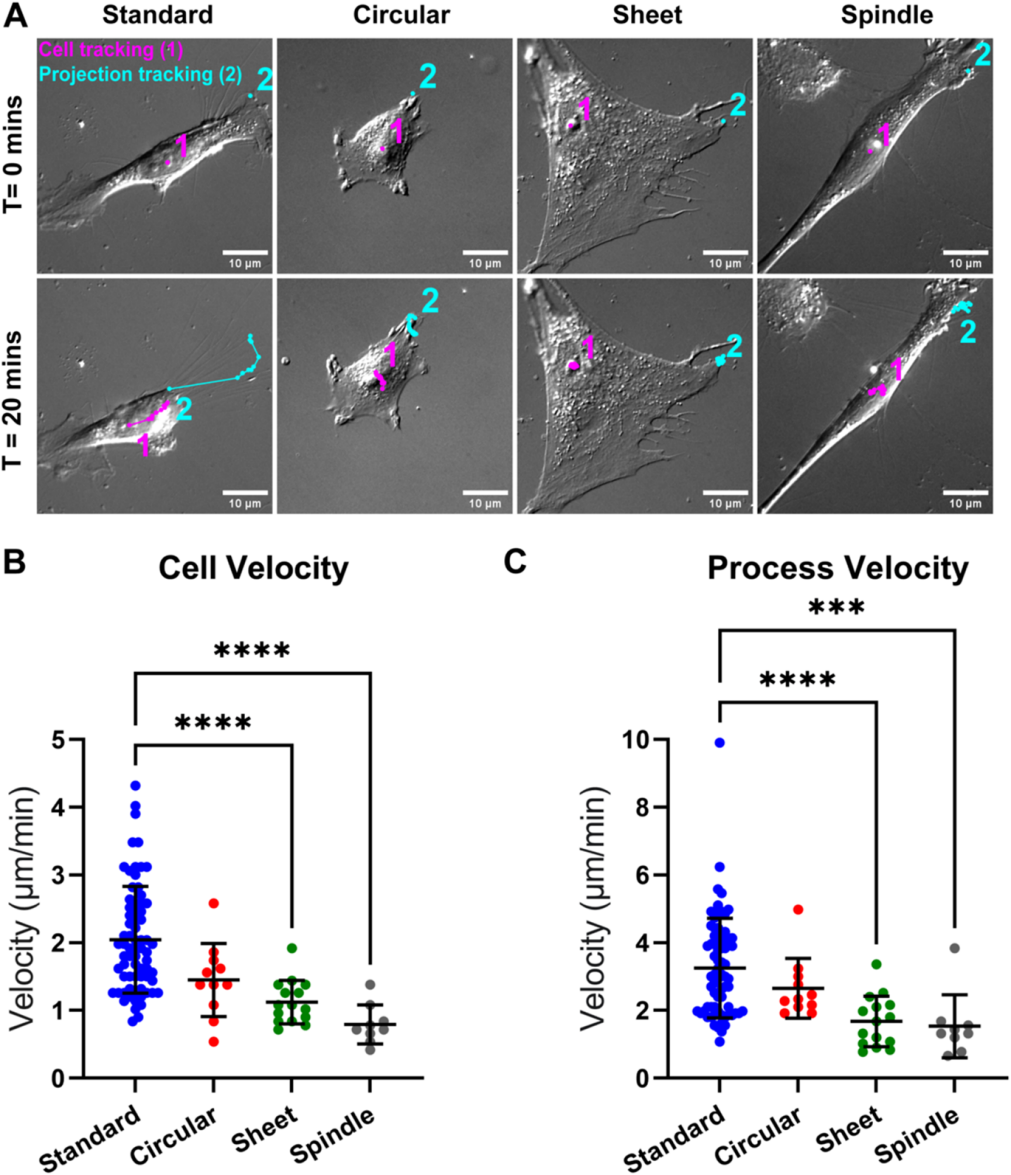
**Pericyte cell velocity and process velocity differs across morphological subtype.** (**A**) Representative DIC images showing morphological subtypes at T = 0 and T = 20 min. Tracks indicate 1 min interval movement of the cell nucleus (pink, 1) and most active membrane projection (cyan, 2). Images from cultures between P6 and P8. (**B**) Mean cell velocity as calculated by the distance of the pink tracks over time in different pericyte morphological subtypes. (**C**) Process velocity in different pericyte morphological subtypes was assessed in the most active process determined by the highest velocity process per cell out of the two primary processes measured as indicated by the cyan tracks. 106 cells were analysed in total. Data points are presented as individual cells overlayed with mean ± SD. Kruskal–Wallis test with Dunn’s multiple comparisons test was used to compare groups. **** *p* < 0.0001, *** *p* < 0.001 compared to Standard

Having noted that over a 2-h period pericytes moved across the well, we next determined whether pericyte motility was associated with morphological subtype. To quantify cell movement, we tracked the nucleus (Fig. 4A), which is known to realign following cell migration (Friedl et al. 2011), for each pericyte. Analysis of cell velocity (Fig. 4B) revealed that standard pericytes were significantly more motile (2.01 ± 0.79 μm/min) compared to both sheet (1.12 ± 0.32 μm/min, *p* < 0.0001) and spindle (0.79 ± 0.29 μm/min, *p* < 0.0001) pericytes, but were not significantly more motile than circular pericytes (1.45 ± 0.54 μm/min, *p* = 0.1007). In addition, we quantified process velocity through tracking the most active of two points on the membrane at the sites of primary projections (Fig. 4C). Process velocity was significantly greater in standard pericytes (2.7 ± 1.31 μm/min) compared to both sheet (1.41 ± 0.64 μm/min, *p* < 0.0001) and spindle (1.42 ± 0.97 μm/min, *p* < 0.0001) pericytes, while there was no difference with circular pericytes (2.30 ± 0.77 μm/min). Together, these data suggest that standard and circular pericytes are motile subtypes, whereas sheet and spindle pericytes have reduced cellular and process motility. As process motility is an important function of both contraction and relaxation of pericytes to modulate blood vessel tone, there may be differences between pericyte subtypes in the responsiveness to vasoactive cues.

### *Pericyte contractility differs across morphological subtype*

**Fig. 5:**
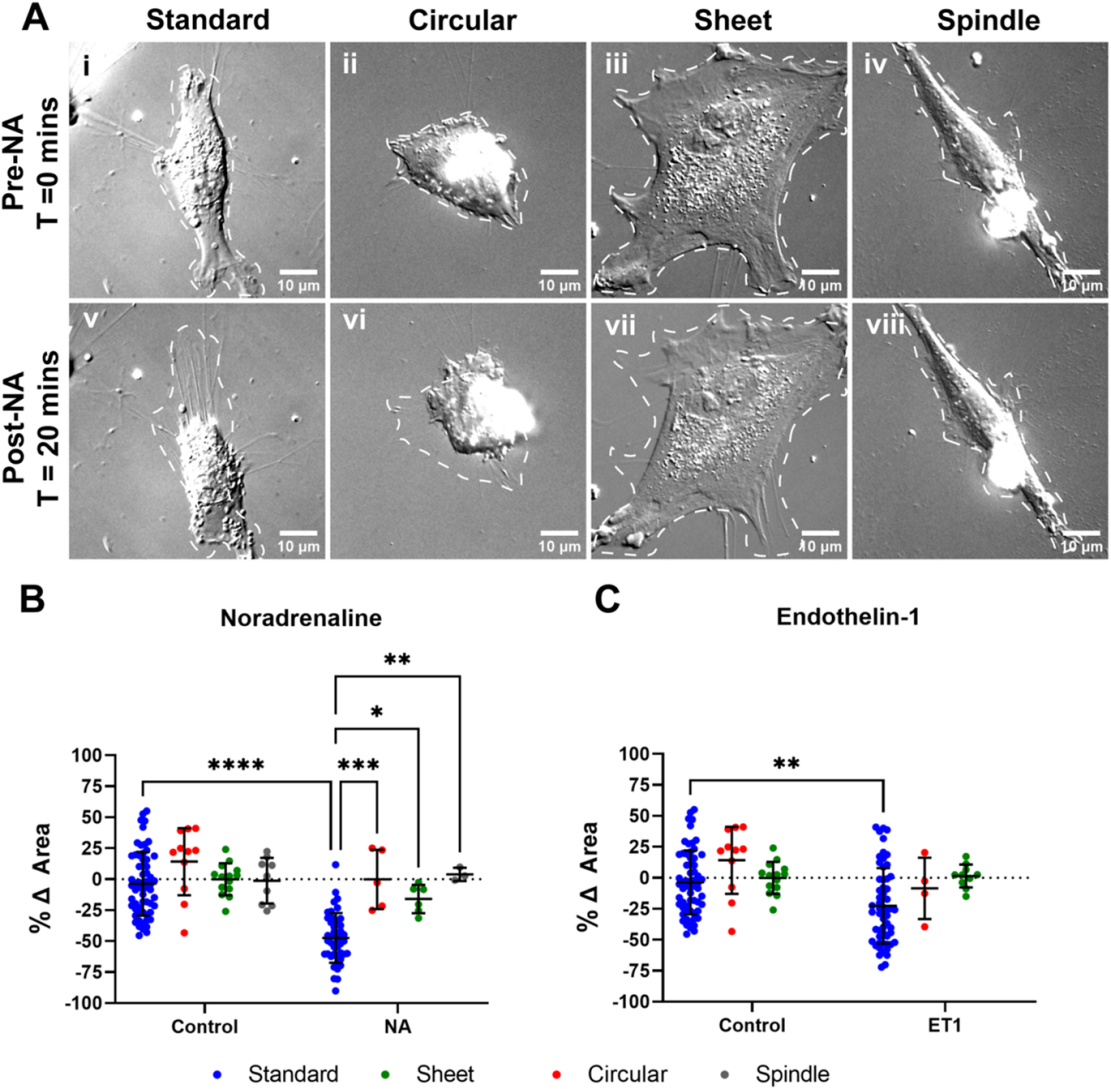
**Standard morphology pericytes contract in response to NA and ET1.** (**A**) Representative panel of DIC images of standard (i), circular (ii), sheet (iii), and spindle (iv) pericytes at T = 0 and T = 20 min treated with NA. The white dotted outline indicates tracing of the cell area. Images from cultures between P6 and P8. (**B**–**C**) Relative change in cell area compared to baseline area following exposure to NA (**B**) and ET1 (**C**). Control indicates treatment with a DMSO vehicle. 233 cells were analysed in total. Data points are presented as individual cells overlayed with mean ± SD. Kruskal–Wallis test with Dunn’s multiple comparisons test was used to compare groups. **** *p* < 0.0001, *** *p* < 0.001, ** *p* < 0.001, * *p* < 0.05 compared to Standard in either control or treatment group

Pericytes have previously been shown to be contractile in vitro (Neuhaus et al. 2017) and their contractility in vivo appears to be restricted to the ensheathing pericytes at the arteriolar end of the capillary bed (Gonzales et al. 2020; Hartmann et al. 2022). Here, after determining morphological subtype differences in pericyte motility, we investigated whether contractility also differed between subtypes. First, we assessed whether pericytes expressed the receptors for the vasoconstrictors NA and ET1. Expression of the NA receptor α_1B_-Adrenergic receptor (α_1B_) was highly expressed in pericytes, while expression of the endothelin A receptor (ET_A_) revealed a more punctate staining pattern (Supplementary Fig. 6A). Following 20-min exposure to the vasoconstrictor NA, standard pericytes responded most strongly through process retraction and a reduction in cell area, while minimal changes occurred in other subtypes (Fig. 5A). Quantification (Fig. 5B) showed that NA significantly reduced cell area in standard pericytes from baseline (− 47.4 ± 20.1%) compared to control treatment (− 3.7 ± 25.7%, *p* < 0.0001; Fig. 5B). There were no significant changes in cell area in circular, sheet or spindle pericytes compared to controls. Similar results were obtained with the vasoconstrictor ET1 (Fig. 5C), whereby standard pericytes had reduced cell area from baseline with ET1 (− 22.6 ± 30.5%) compared to control conditions (− 3.7 ± 25.7%, *p* = 0.0016), with no significant change in other subtypes (Fig. 5C). The extent of pericyte contraction was greater with NA compared to ET1. To confirm that cell contraction was not a result of the initiation of cell death, we performed a cell death assay, which showed that exposure to NA and ET1 did not lead to a significant increase in cell death over 1 h (Supplementary Fig. 6B). To determine whether standard pericytes could also relax, adenosine, a known vasorelaxant, was administered following pericyte contraction with NA. Exposure of pericytes previously contracted with NA to adenosine revealed a restoration above baseline cell area, indicating pericytes could also relax (Supplementary Fig. 7). These data suggest that standard pericytes are the most responsive to vasoactive cues.

### *Pericyte αSMA expression differs across morphological subtype*

**Fig. 6:**
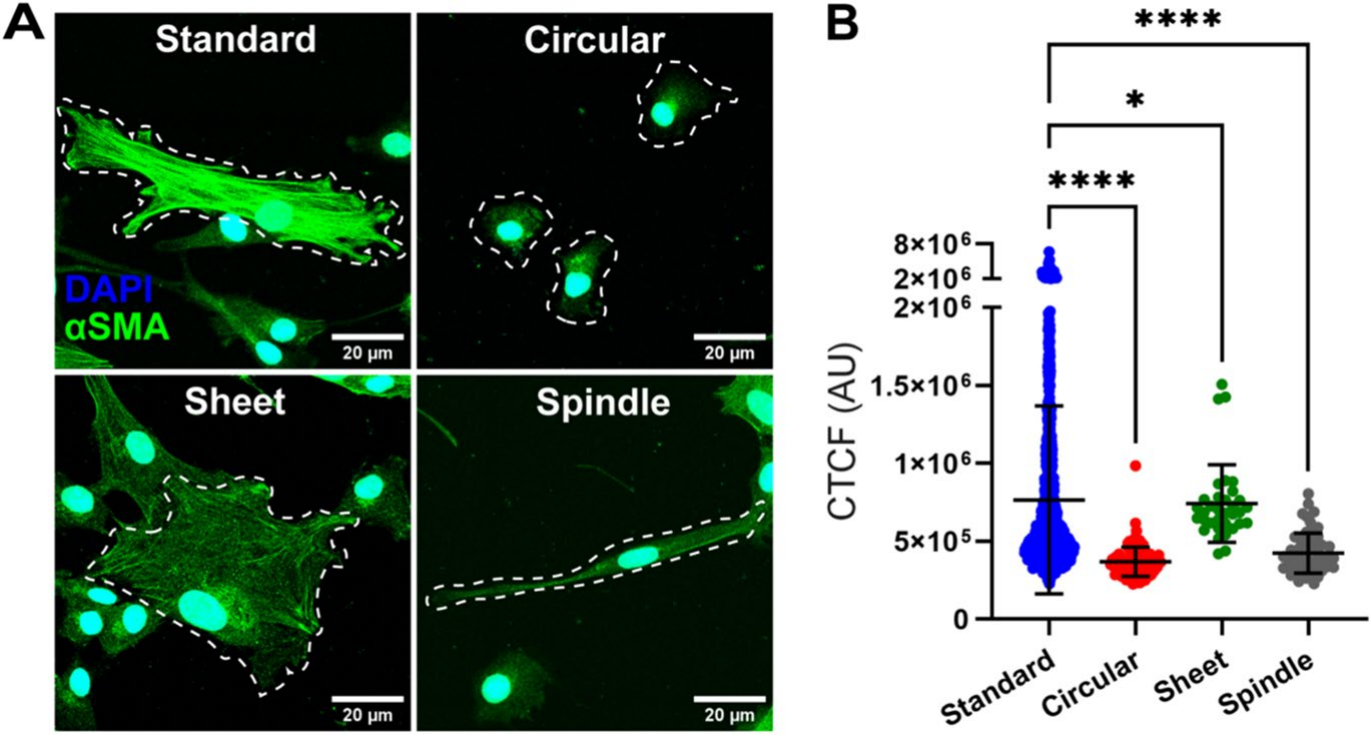
**αSMA expression in pericytes varies across morphological subtype.** (**A**) Representative confocal microscopy images of pericyte subtypes with αSMA-positive (green) and DAPI-positive nuclei (blue). White dotted outlines represent cell membrane boundaries. Images from P6 cultures. (**B**) Quantification of corrected total cell fluorescence (CTCF) of αSMA expression between pericyte subtypes. 1219 cells were analysed in total. Data points are presented as individual cells overlayed with mean ± SD. Kruskal–Wallis test with Dunn’s multiple comparisons test was used to compare groups. **** *p* < 0.0001, * *p* < 0.05 compared to standard

One of the key proteins involved in cytoskeletal rearrangement during cell contraction is αSMA (Hinz et al. 2001; Wang et al. 2006). Given that we observed differences in the extent of contractility between pericyte subtypes, we hypothesised that this may be due to differing expression of the key cell contractility protein αSMA. Immunostaining showed HBVP cultures had widespread expression of αSMA protein and we found variations in αSMA staining intensity between cells and between subtypes (Fig. 6A). We used CTCF to quantify fluorescence intensity of αSMA to determine any differences in between subtypes. Standard pericytes had the greatest level of αSMA expression (7.64 × 10^5^ ± 6.03 × 10^5^ AU) which was significantly higher than both circular (3.68 × 10^5^ ± 9.32 × 10^4^ AU, *p* < 0.0001) and spindle (4.23 × 10^5^ ± 1.28 × 10^5^ AU, *p* < 0.0001) pericytes. Standard pericytes also had higher αSMA expression than sheet pericytes (7.42 × 10^5^ ± 2.49 × 10^5^ AU, *p* = 0.0170), though levels were still moderately high in sheet pericytes compared to circular and spindle (Fig. 6B). Interestingly, sheet pericytes also had some level of contraction compared to baseline following NA administration (− 16.00 ± 11.39%; Fig. 5B). These data suggest that the differential contractile capabilities of pericyte subtypes may be associated with each subtype’s αSMA expression.

## Discussion

After close examination of HBVP by DIC microscopy, here we report five major subtypes of pericyte morphology in culture. The majority of pericytes in culture (around 70%) exhibited ‘standard’ morphology and the remaining four morphologies were in similar proportions to each other (5–12% of all cells). Cell tracing analysis identified key shape descriptors that could describe each morphology, and cell tracing technology could be used to distinguish morphological subtype of pericytes to ensure consistency of cultures throughout all experiments. While heterogeneity of pericytes on blood vessels in vivo is thought to be dependent on location of the vascular tree (Berthiaume et al. 2018b; Grant et al. 2019), pericytes cultured in a monolayer in vitro do not have tubular structures to adhere to or in vivo cues to interact with such as factors released from endothelial cells (Smyth et al. 2018), suggesting that other factors may produce the morphological and functional differences of pericytes within a culture. The development of 3D co-cultures mimicking a tubular blood vessel structure could lead to similarities in pericyte morphologies between in vitro and in vivo studies (Campisi et al. 2018; Kim et al. 2015). Here we highlight that differential pericyte morphology in cultured HBVPs is associated with differences in motility, contractility and protein expression. Importantly, standard pericytes were the most contractile subtype with the greatest expression of αSMA, highlighting the importance of protein expression to function within individual cells of pericyte cultures. Therefore, the implications of cellular heterogeneity within cultures need to be considered when assessing pericyte function.

Passage number is critical for the use of pericytes in culture as phenotype and gene expression may change over time, as has been shown previously with placental pericytes (Shiwen et al. 2009). Between passages 3 and 9, the proportions of each subtype remained similar, suggesting that morphological heterogeneity displayed within cultures was not related to cell passage. Passage 9 represents approximately 18 population doublings which supports the recommendation from ScienCell for the use of HBVPs up to 15 population doublings. However, from passage 10 the proportion of standard pericytes significantly reduced while the proportion of spindle cells increased. Spindle pericytes appeared to be less motile and did not proliferate as readily as other morphologies. Interestingly, pericytes in the aged mouse brain have reduced function including diminished remodelling capacity and dynamic control over blood vessels in vivo (Berthiaume et al. 2022). Cell morphology changes have been observed with greater passage number for other cell types (Neumann et al. 2010; Yang et al. 2018) restricting experiments to specific passage number containing optimal cells. Our RNAseq data highlighted a number of differentially expressed genes between passages 5 and 9, many of which were involved in extracellular matrix organization, metabolism and biosynthesis, and symporter activity. This indicates that to maintain consistent cultures, experiments should be performed in HBVP within defined passage numbers at similar cell densities.

Both media and confluency of cells may have influenced morphology and function of cultured pericytes. Our studies on HBVP were conducted using complete pericyte media sourced from ScienCell, whose formulation is proprietary. A previous study has assessed the phenotype and neuroinflammatory responses of primary human pericytes exposed to pericyte media from ScienCell compared to DMEM/F12 media (Rustenhoven et al. 2018). This paper noted differences in pericyte proliferation, morphology, protein expression, and phagocytic and migratory ability between both media types. These phenotypic changes were assessed between cultures, and do not account for any pericyte heterogeneity within cultures as observed in our study. Our rat brain pericyte analysis showed that similar morphological heterogeneity exists in cultures that were grown in DMEM-based media, and it will be important for future studies to ensure that morphological and functional heterogeneity exists in different media formulations within a culture. In addition, our experiments were conducted at approximately 60–80% confluency to enable individual pericyte visualization using single cell imaging. While most studies appear to use a confluency > 80%, a very high confluency can lead to contact inhibition which can arrest cell growth (Seluanov et al. 2009) and initiate cell death due to depletion of nutrients (Busschots et al. 2015). Therefore, it is possible that the heterogeneity in both morphology and function of cultured pericytes may change in a high confluency environment.

While the proportion of morphological subtypes changed with later passage, imaging over a 2-h period showed that morphology of individual cells did not change quickly under basal conditions. Single pericyte ablation studies have shown that it takes many days for pericytes to morphologically adapt to cover endothelial cells on the capillary, suggesting morphological change of in vivo pericytes is a slow process (Berthiaume et al. 2018a). Tracking cell and process velocity over time showed that in culture, standard pericytes were the most active, with extension and contraction of their processes. This aligns with capillary pericytes in vivo where extension and retraction of processes have been shown to aid in the coverage of the vasculature and sensing of molecules from within the circulation and the brain parenchyma (Berthiaume et al. 2022). It is possible that differences in morphology could represent different states of the cell cycle. It is known that as mammalian cells enter different stages of the cell cycle, their morphology changes (Thery and Bornens 2006). Our morphology analysis highlighted balled cells as cells about to undergo cell division, and we identified that standard, circular and sheet cells all underwent cell division. Further research is needed to confirm whether morphological subtypes of pericytes represent different stages of the cell cycle.

One of the key functions of pericytes in the brain is to regulate cerebral blood flow (Hall et al. 2014), but it is thought that only ensheathing pericytes on the arteriolar end of the capillary bed are able to contract and relax capillaries (Attwell et al. 2016). Previous in vitro and ex vivo studies have shown that pericytes can contract or relax in response to vasoactive mediators (Hall et al. 2014; Neuhaus et al. 2017), but knowledge on whether the pericyte response to these mediators varies with morphological subtype is limited. Here, we showed that standard pericytes contracted most strongly in response to both NA and ET1 compared to other subtypes. Given that standard pericytes make up the majority subtype in HBVP cultures, this may explain why contraction and relaxation still occurred in whole-well assays (Neuhaus et al. 2017). However, some sub-population differences within cultured pericytes for Ca^2+^ release (Halaidych et al. 2019) and cell contraction (Heyba et al. 2019) were observed following ET1 stimulation. We then explored whether there were differences in αSMA expression between morphological subtypes. αSMA is a critical protein mediating the contraction of vascular smooth muscle cells (Wang et al. 2006), and has been shown to be expressed at differing levels in pericytes in vivo (Attwell et al. 2016). Our subtype analysis revealed that pericytes with standard morphology expressed αSMA most strongly, which was associated with the most profound contraction. Other morphological subtypes expressed αSMA to a lower level but did not exhibit contraction in response to NA or ET1. Future studies should look at vasoactive mediator receptor expression in the different pericyte subtypes as differential NA or ET1 receptor expression levels may lead to the differences in contractility we observed. One limitation of using cell retraction, a method to measure pericyte contractility which has been used previously (Kelley et al. 1987; Neuhaus et al. 2017), is cell retraction or loss of cell area could be due to other factors such as apoptosis or trypsinization. We have attempted to overcome this by showing no cell death occurred with NA or ET1 administration, and pericytes could actively relax (increase their cell area) in response to adenosine. Other contraction assays would be needed to confirm these effects.

There were a few other limitations to our study. Our initial classification of morphological subtypes of pericytes was performed manually, but the shape descriptors following cell tracing revealed clear differences between subtypes. We also only imaged cells up to 2 h, and longer imaging times could determine if changes in morphology occur more frequently. Additional studies may also use FACS to distinguish phenotypic heterogeneity in pericytes similar to that previously used for cell cycle analysis in HEK293 cells (Kage et al. 2021). In addition, single cell transcriptomics could be applied on FACS sorted cells to uncover molecular characteristics of each morphological subtype.

With the current evidence, it is difficult to conclude which in vitro subtype of pericyte corresponds to the subtype of pericyte in vivo. Brain pericytes on capillary beds have various morphology, protein and mRNA expression, and functions depending on their location in the vascular tree. For example, expression of αSMA is thought to be associated with contractile properties in ensheathing pericytes (Attwell et al. 2016), with limited expression in other pericyte subtypes (Vanlandewijck et al. 2018). However, Smyth et al. (2018) showed some αSMA expression on junctional pericytes in vivo, while Alarcon-Martinez et al. (2018) showed that capillary pericytes do express αSMA which rapidly depolymerizes during tissue fixation thus evading detection by immunolabeling. While we showed that HBVPs had heterogeneous αSMA expression, expression of αSMA in in vitro pericytes has been shown previously, which may be due to upregulation of αSMA expression in culture conditions (Heyba et al. 2019; Smyth et al. 2018). Here, we only assessed whether pericyte subtypes had differing motility and contractility, and further analysis of other pericyte functions such as BBB integrity, immune activity and susceptibility to cell death would need to be carried to determine the full scope of functional heterogeneity between morphological subtypes of pericytes, and whether there was an association with an in vivo subtype.

In conclusion, we have identified distinct morphological subtypes in pericyte cultures, which display different behaviours. The predominant standard morphology had the greatest cell and process motility, and contractile response to vasoactive mediators. Morphological diversity within cell culture systems is important to consider when performing assays, considering that the proportion of morphological subtypes in pericyte cultures change as passage number increases, which may lead to different functional properties. Given that pericytes in the brain have substantial morphological and functional differences depending on where they sit within the vascular tree, it is important to consider and understand pericyte heterogeneity in vitro in order to inform brain pericyte biology.

### Supplementary Information

Below is the link to the electronic supplementary material.

Supplementary file1 (DOCX 11448 KB)

## Data Availability

The datasets generated during and/or analysed during the current study are available from the corresponding author on reasonable request.
