# Supplementary material for: Brain pericytes in culture display diverse morphological and functional phenotypes
Source: Cell Biol Toxicol. 2023 Jun 16;39(6):2999–3014. doi: 10.1007/s10565-023-09814-9 (PMC10693527; doi:10.1007/s10565-023-09814-9)
Supplement: Supplementary file 1 — Supplementary file1 (DOCX 11448 KB) [file 10565_2023_9814_MOESM1_ESM.docx]

Brain pericytes in culture display diverse morphological and functional phenotypes

Lachlan S. Brown^1^, Natalie E. King^1^, Jo-Maree Courtney^1^, Robert J. Gasperini^1^, Lisa Foa^1,2^, David W. Howells^1^, Brad A. Sutherland^1^

1. Tasmanian School of Medicine, College of Health and Medicine, University of Tasmania, Hobart, Tasmania, Australia

2. School of Psychological Sciences, College of Health and Medicine, University of Tasmania, Hobart, Tasmania, Australia

**Correspondence:**

A/Prof Brad A. Sutherland,

Level 4 Medical Sciences Precinct,

17 Liverpool St,

Hobart, TAS 7000

Australia

Email: Brad.sutherland@utas.edu.au

# **Supplementary Methods**

## ***Immunocytochemistry for pericyte markers and vasoactive receptors***

Immunocytochemistry was performed using the same methods outlined in the main manuscript, with the addition of the following primary antibodies: 1:500 goat anti-PDGFRβ, R&D Systems, AF1042; 1:500 rabbit anti-CD13, Abcam, ab108310; 1:500 rabbit anti-NG2, Chemicon, Merck, AB5320; 1:500 rabbit anti-CD31, ThermoFisher, 14-0311-82; 1:500 rabbit anti-ADRA1B, Abcam, ab169523; 1:500 rabbit anti-ET1AR, Abcam, ab178454), all diluted in Antibody Diluent (Dako, #S080983-2) and applied overnight in a humidified chamber at 4°C. Cells were imaged using the same methods outlined in the main manuscript.

## ***Primary rat brain pericyte culture***

Primary pericyte cultures were isolated and prepared from 1-2 month old Sprague-Dawley rats as previously described (Redzic et al., 2015), which is a modified protocol without the puromycin-containing medium to limit pericyte growth (Abbott et al., 2012). Briefly, brain endothelial cells and pericytes were grown together in a mixed primary culture from capillary fragments, and after 2-3 weeks following plating, at which time pericytes outgrow endothelial cells, cells were passaged and plated on uncoated flasks. Pericytes were grown in DMEM media with growth supplements (including selenium, insulin, transferrin, vitamin C, glutathione and basic fibroblast growth factor) and imaged using a Leica inverted microscope.

## ***RNA sequencing and analysis***

HBVP at passages 5 and 9 were cultured in 6-well plates, and RNA was extracted with RNeasy Mini Kit (Qiagen, #74104) as per manufacturer’s instructions. RNA quality was assessed using TapeStation 4200 (Agilent Technologies) and only samples with a RIN>8 were used. DNA-free RNA samples were then sent to the Australian Genome Research Facility for sequencing. Following library preparation for each sample, RNA sequencing (100bp single-end run) was carried out using an Illumina NovaSeq platform. Following demultiplexing and quality control, the cleaned sequence reads were aligned against the *Homo sapiens* genome (build version HG38) using the STAR aligner (v2.7.10a) (Dobin et al., 2013). Raw gene counts were generated and transcripts were assembled with the StringTie tool (v2.1.4) (Pertea et al., 2015). Differential gene expression analysis was conducted using DESeq2 (v1.36.0) (Love et al., 2014) in R (v4.2.1) with an adjusted p-value (false discovery rate) <0.05 and absolute log_2_(fold-change) > 1 considered to be differentially expressed. Gene ontology enrichment analysis was conducted using clusterProfiler (v4.4.4) (Wu et al., 2021).

## ***Cell death assay***

A PI-Hoechst assay was used to determine whether pericytes were dying in response to addition of vasoactive mediators. The extent of PI-labelled nuclei indicates the level of cell death as seen in previous literature (King et al., 2022). HBVP at passage 6 were cultured into 96 well plates and grown to 75% confluence. Live imaging buffer was prepared [Hanks’ Balanced Salt Solution (no Ca^2+^, Mg^2+^, phenol red), 1X, Thermofisher Scientific, #14185052; HEPES buffer solution, 15mM, Thermofisher, #15630080; Glucose solution, 30mM, Sigma Aldrich, #G8769; MgCl_2_, 1mM, Sigma Aldrich, #M4880; CaCl_2_, 2mM, Sigma Aldrich, #C5670; made up to volume with MilliQ H_2_0 and pH 7.4]. Media was replaced with imaging buffer containing: NA, 10μM, Sigma, A7257; ET1, 500nM, Sigma, E7764; imatinib, 500μM, Sapphire Bioscience, A10259, as a positive control for cell death (King et al., 2022); or DMSO, 0.1% v/v, as a vehicle control. Cells were incubated for 1 hour, then media was replaced with imaging buffer containing Hoechst for a final dilution of 1:1000 from stock (Invitrogen, #H3570), and PI (0.02mg mL^-1^, Sigma Aldrich, #P4864) for 10 minutes. Cells were imaged in the blue (DAPI Ex. 377nm, Em. 447nm) and red (Texas Red Ex. 558nm, Em. 583nm) fluorescence channels at 10x magnification from the centre of each well using a Cytation 5 Cell Imaging Multi-Mode Reader (Biotek). Images were processed on Gen5 software (Biotek) and exported to ImageJ. An ImageJ macro was created to determine total and dead cell number which separated the Hoechst and PI channels for each image, thresholded for positive cell bodies, removed outliers and counted the number of positive nuclei using the analyse particles function. Dead cell percentage were calculated as the number of PI-positive nuclei divided by the total number of nuclei and represented as the percentage of total cells.

# **Supplementary References**

Abbott, N. J., et al., 2012. An improved in vitro blood-brain barrier model: rat brain endothelial cells co-cultured with astrocytes. Methods Mol Biol. 814; 415-30. 10.1007/978-1-61779-452-0_28

Dobin, A., et al., 2013. STAR: ultrafast universal RNA-seq aligner. Bioinformatics. 29; 15-21. 10.1093/bioinformatics/bts635

King, N. E., et al., 2022. Pharmacological PDGFRbeta inhibitors imatinib and sunitinib cause human brain pericyte death in vitro. Toxicol Appl Pharmacol. 444; 116025. 10.1016/j.taap.2022.116025

Love, M. I., et al., 2014. Moderated estimation of fold change and dispersion for RNA-seq data with DESeq2. Genome Biol. 15; 550. 10.1186/s13059-014-0550-8

Pertea, M., et al., 2015. StringTie enables improved reconstruction of a transcriptome from RNA-seq reads. Nat Biotechnol. 33; 290-5. 10.1038/nbt.3122

Redzic, Z. B., et al., 2015. Differential effects of paracrine factors on the survival of cells of the neurovascular unit during oxygen glucose deprivation. Int J Stroke. 10; 407-14. 10.1111/ijs.12197

Wu, T., et al., 2021. clusterProfiler 4.0: A universal enrichment tool for interpreting omics data. Innovation (Camb). 2; 100141. 10.1016/j.xinn.2021.100141

# **Supplementary Figures**


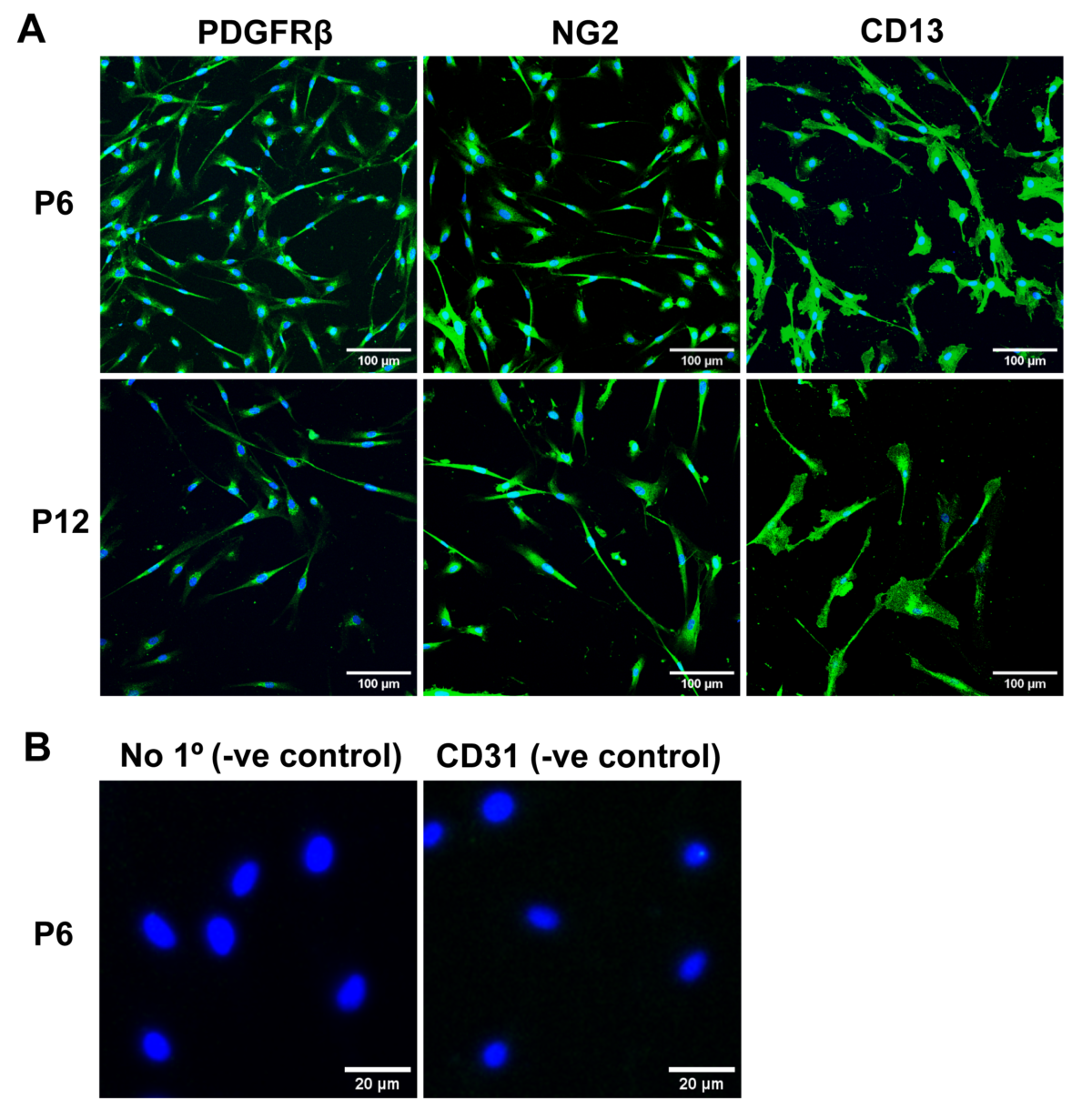


**Supplementary Figure 1. Cultured human brain vascular pericytes express classical pericyte proteins. *(A)*** *Fluorescent microscopy images of HBVP from passage 6 (P6) and passage 12 (P12) cultures immunolabelled with the nuclear stain DAPI (blue) and antibodies (green) against pericyte markers PDGFRβ, NG2, and CD13.* ***(B)*** *No primary antibody (No 1º) negative control, and CD31 (endothelial cell marker) negative control. Scale = 100μm (A) and 20μm (B).*


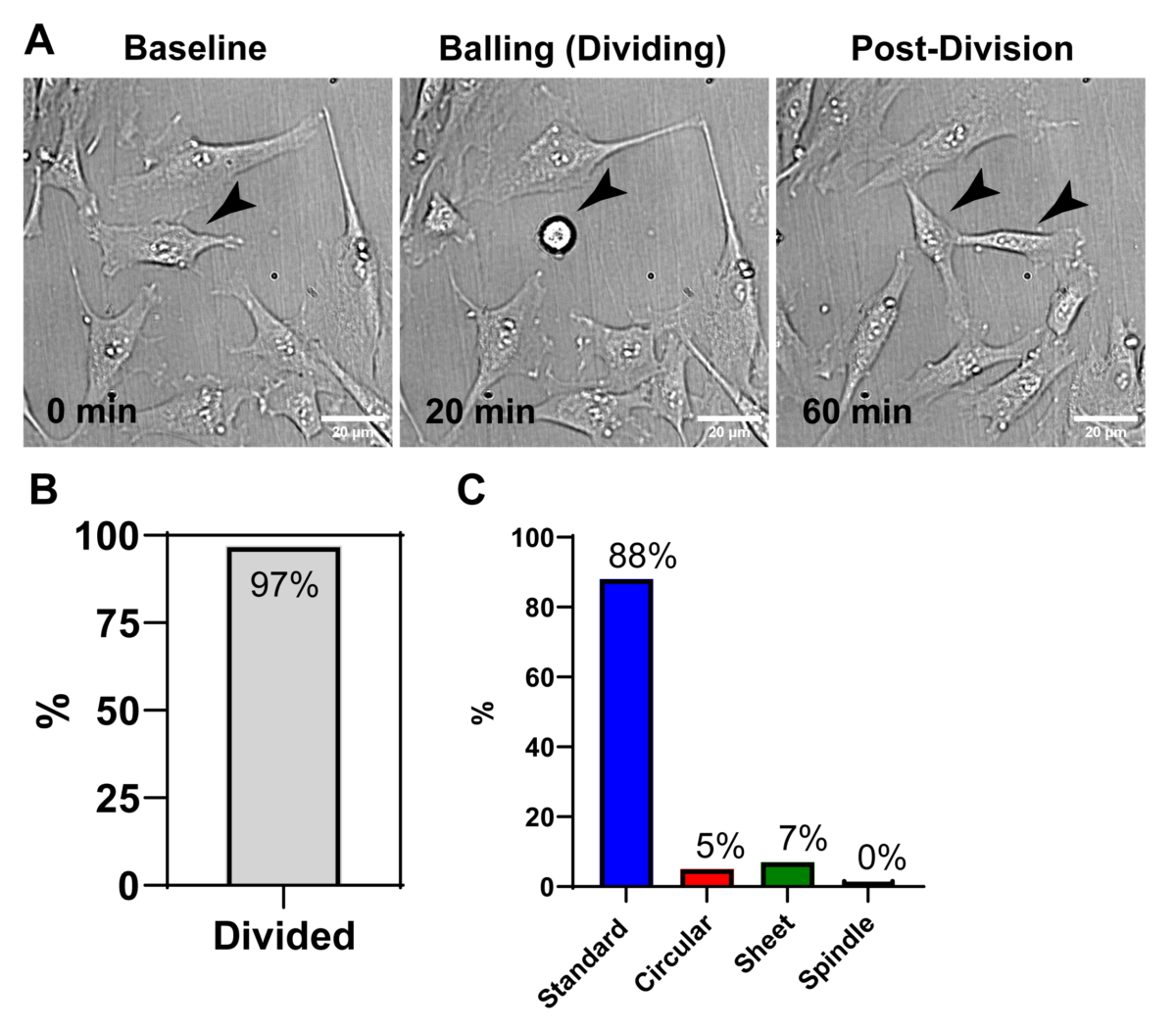


**Supplementary Figure 2. Balling morphology represents pericytes undergoing cell division. *(A)*** *Representative DIC images of pericyte at baseline, balling (dividing), and post-division. Black arrows indicate cell prior to balling, balling, and both daughter cells post-division. Images from P6 cultures.* ***(B)*** *Quantification of number of balling cells that divided within two hours. N=100 cells analyzed.* ***(C)*** *Percentage of* *each classified morphology of the parent cell prior to balling (N=100) in Supp Fig 2B.*


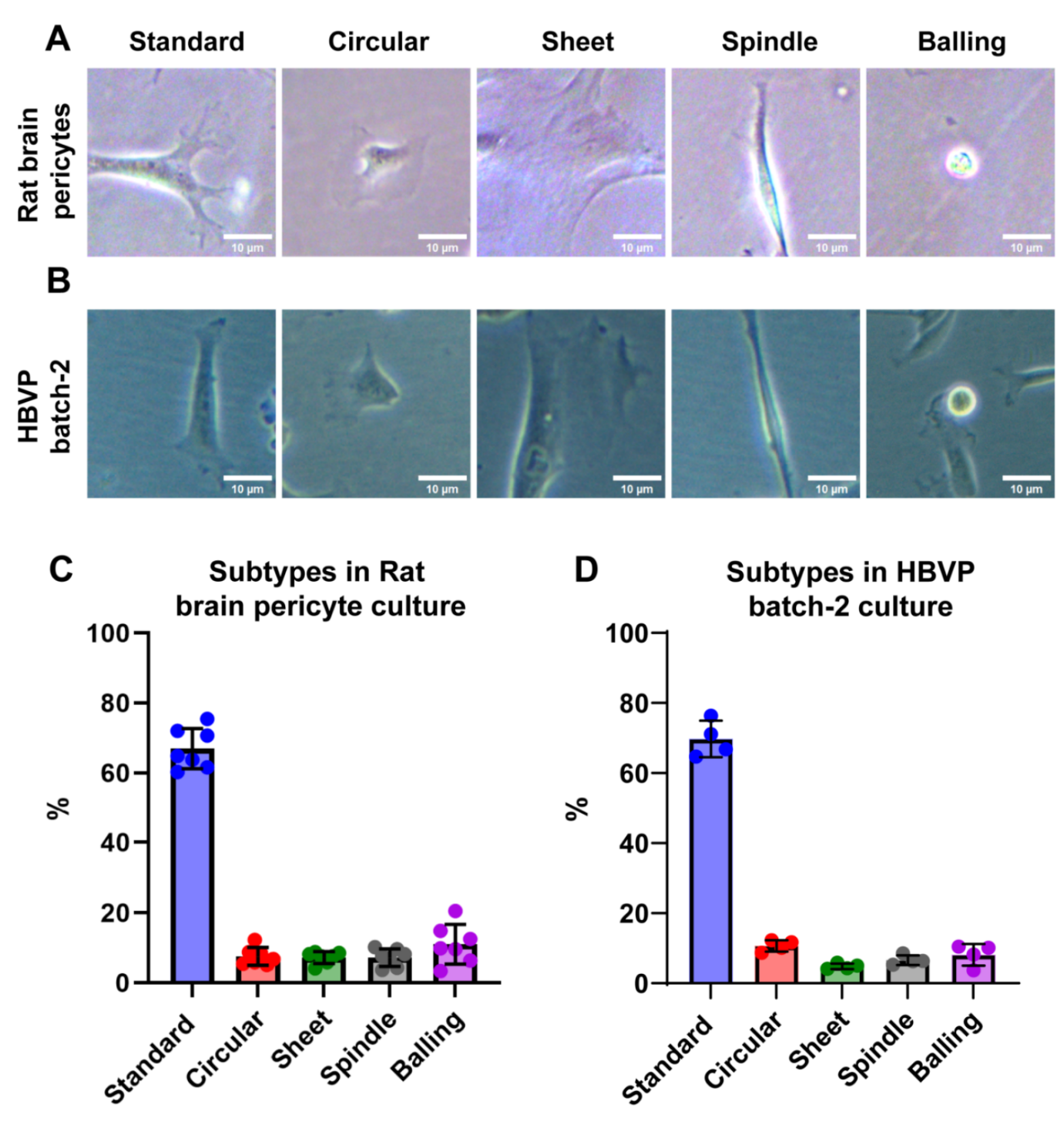


Supplementary Figure 3. In vitro rat brain pericytes exhibit morphological heterogeneity similar to HBVPs. (A-B) Representative brightfield images of five different morphologies observed in primary rat brain pericyte cultures (A) and an additional batch of HBVP (B) distinct from the batch presented in Fig 1: standard, circular, sheet, spindle, and balling morphologies. Images from rat brain pericytes at passage 2 post-isolation (see Supplementary Methods). Images from HBVPs were from passage 6. (C-D) Percentage of each morphology within cultures was calculated. N = 7 cultures and 73-157 cells analysed per culture (742 total cells analyzed) (C), and N = 4 cultures and 208-322 cells analysed per culture (1115 total cells analysed) (D). Data points are presented as individual cultures overlayed with mean ± SD.


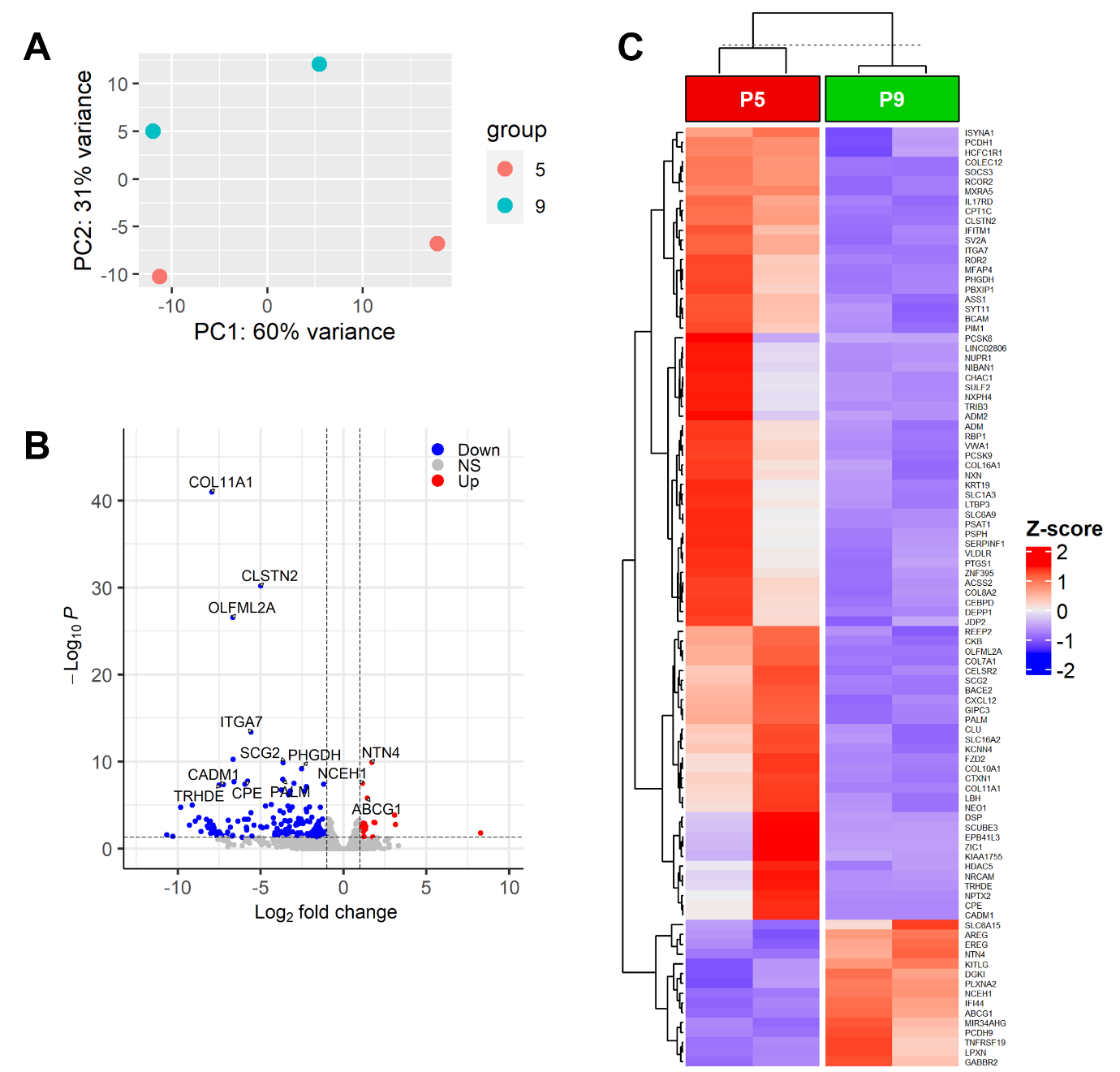


Supplementary Figure 4. Gene expression changes in pericytes between passage 5 and 9. (A) Principal Component (PC) analysis indicates PC1 and PC2 account for 91% variance and cells from passage 5 and passage 9 segregate based on PC2. (B) Volcano plot showing upregulated genes in red and downregulated genes in blue. (C) Heat map of RNA-seq data outlining the expression of the 96 significant differentially expressed genes between passage 5 and passage 9. ­

*
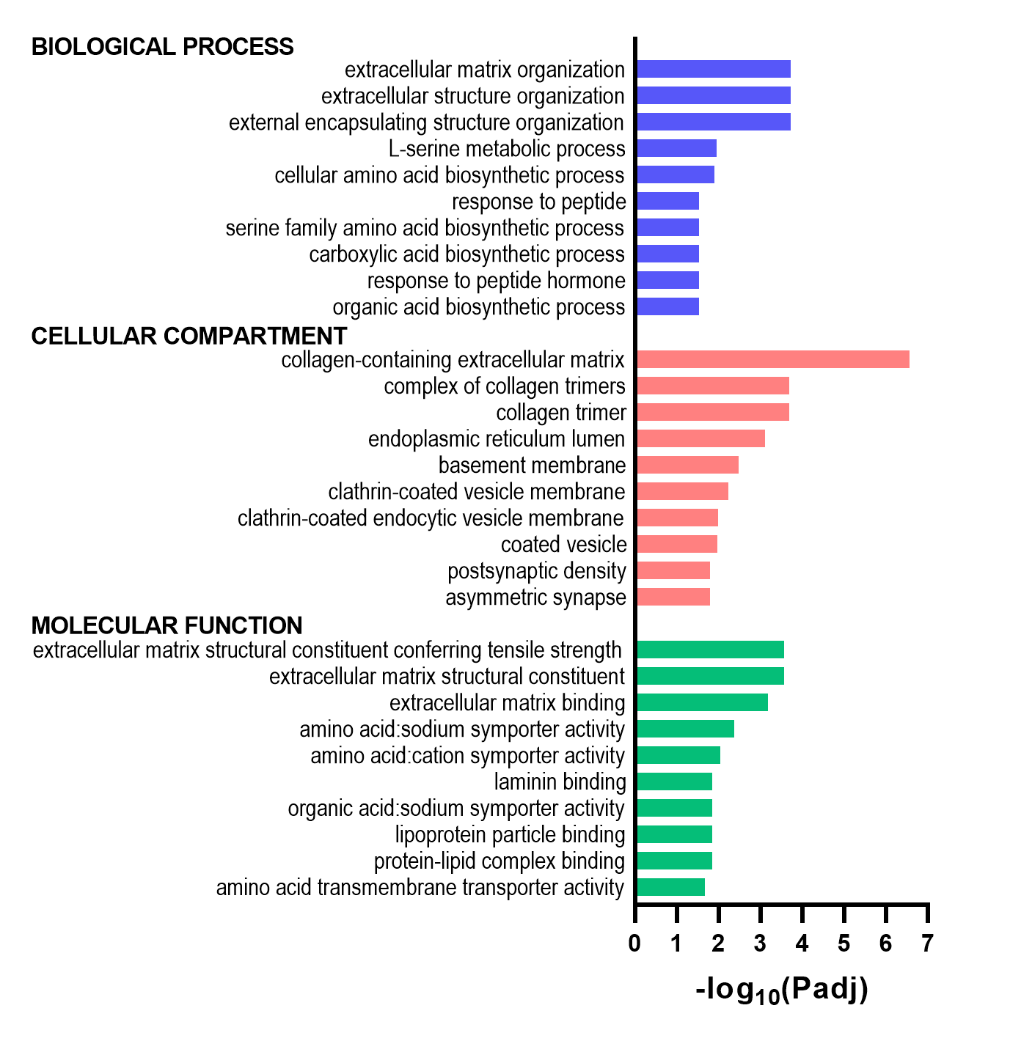
*

Supplementary Figure 5. Functional enrichment analysis. Top 10 enriched gene ontology (GO) terms within categories: biological process, cellular component, and molecular function for significant differentially expressed genes between passage 5 and passage 9.

**
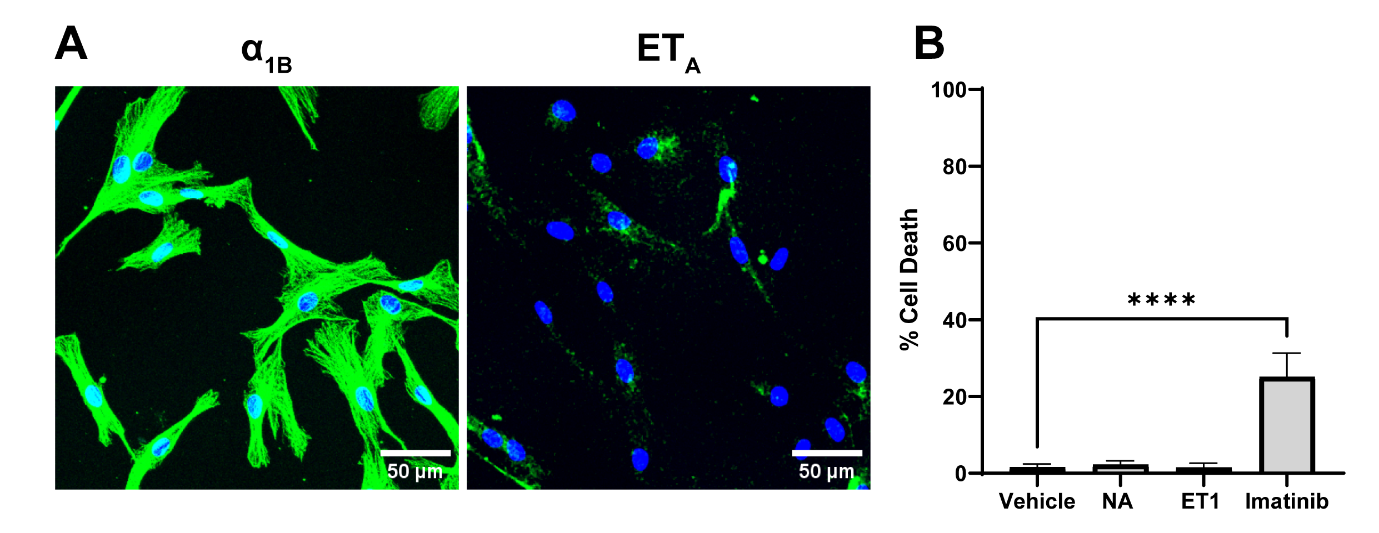
Supplementary Figure 6. Cultured human brain vascular pericytes express NA and ET1 receptors and do not undergo cell death with vasoconstrictors.** ***(A)*** *Fluorescent microscopy images of HBVP from passage 6 (P6) cultures immunolabelled with the nuclear stain DAPI (blue) and antibodies (green) against α-1B adrenergic receptor (α_1B_) and endothelin receptor type A (ET_A_).* ***(B)*** *Percentage cell death after 1 hour treatment with NA, ET1 or imatinib (a known inducer of pericyte death), calculated by number of dead cells (PI-positive) divided by the total cells (Hoechst-positive). Data are presented as mean ± SD and are calculated from 2x2mm stitched images from the centre of wells in a 96 well plate. N = 8 wells per treatment. Data from P6 cultures. Ordinary One-Way ANOVA with Dunnett’s multiple comparisons test was used to compare groups.*

**
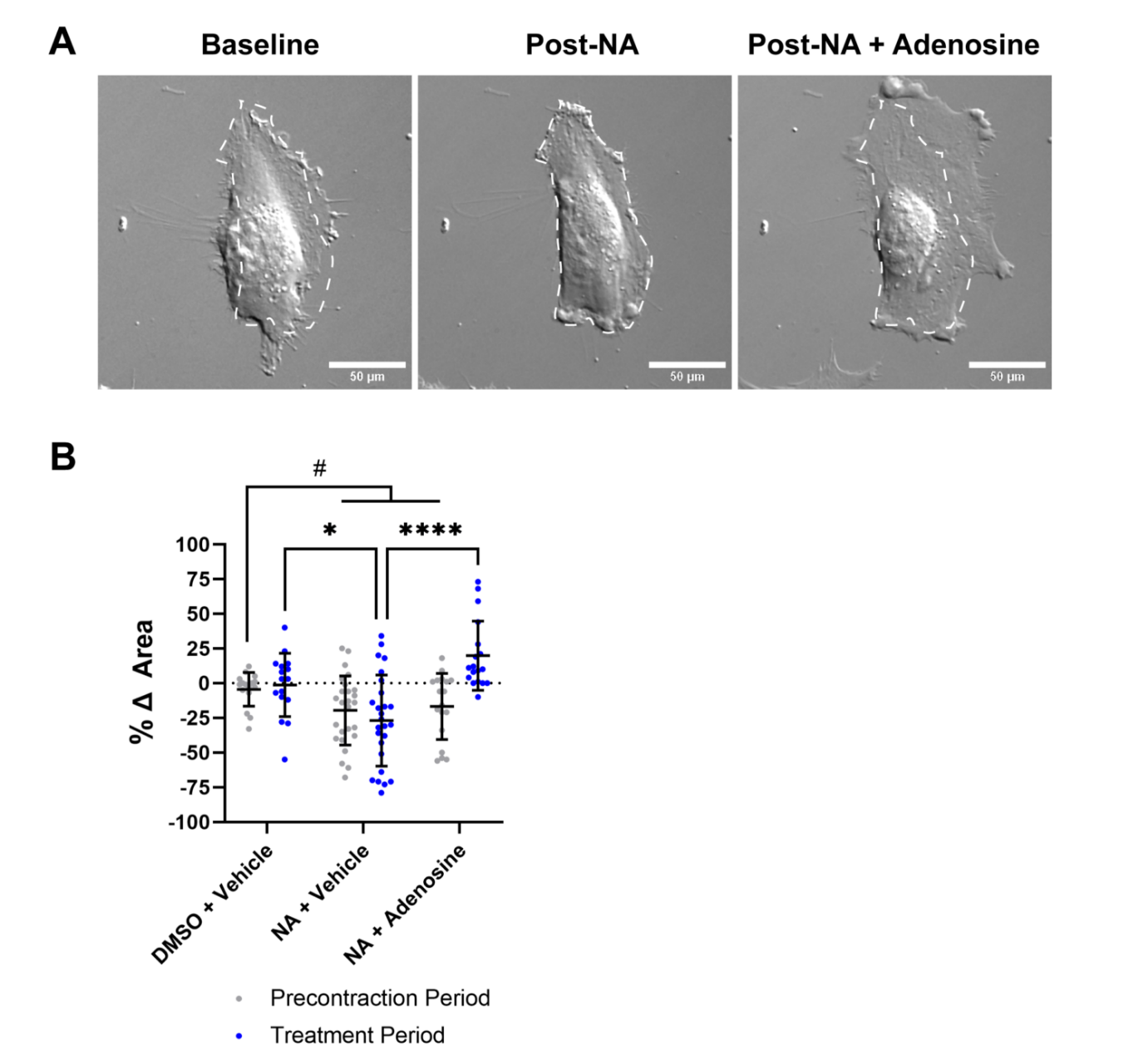
**

**Supplementary Figure 7. Adenosine induces relaxation of standard morphology pericytes after pre-contraction with noradrenaline.** ***(A)*** *Representative DIC images of HBVP at baseline (baseline; T = 0 mins), post treatment with noradrenaline (post-NA; T = 10 mins), and post treatment with noradrenaline and adenosine (NA + adenosine; T = 60 mins). The white dashed outline indicates tracing of the cell area following NA treatment overlayed over the other time points. Image from P6-8 culture.* ***(B)*** *Relative change in cell area compared to baseline area. Pre-contraction period was 10 mins and cells were exposed to either DMSO vehicle (DMSO) or noradrenaline (NA) to induce pre-contraction. Cells were then exposed to an additional treatment of DMSO (vehicle) or adenosine for a further 50 mins. Data points are presented as individual cells overlayed with mean ± SD. Number of individual cells analysed for each group: DMSO + vehicle (N = 16); NA + vehicle (N = 26); NA + adenosine (N = 18). 60 cells were analyzed in total. Two-way ANOVA with Tukey’s multiple comparisons test was used to compare groups. **** p < 0.0001,* ** p < 0.05. Mann-Whitney test was used to compare DMSO + vehicle to grouped NA + vehicle and NA + AD as these groups had the same treatment over the pre-contraction period (NA). # p < 0.05.*
